# The Association of Mindful Parenting with Glycemic Control and Quality of Life in Adolescents with Type 1 Diabetes: Results from Diabetes MILES—The Netherlands

**DOI:** 10.1007/s12671-016-0565-1

**Published:** 2016-07-13

**Authors:** Inge J. P. Serkel-Schrama, Jolanda de Vries, Anke M. Nieuwesteeg, Frans Pouwer, Ivan Nyklíček, Jane Speight, Esther I. de Bruin, Susan M. Bögels, Esther E. Hartman

**Affiliations:** 1 Department of Child and Adolescent Psychiatry, St. Elisabeth Hospital, Tilburg, The Netherlands; 2 Department of Medical Psychology, St. Elisabeth Hospital, Tilburg, The Netherlands; 3 Center of Research on Psychology in Somatic diseases (CoRPS), Department of Medical and Clinical Psychology, Tilburg University, Tilburg, The Netherlands; 4 Department of Developmental Psychology, Tilburg University, Tilburg, The Netherlands; 5 School of Psychology, Deakin University, Geelong, VIC Australia; 6 The Australian Centre for Behavioural Research in Diabetes, Diabetes Victoria, Melbourne, VIC Australia; 7 Research Institute Child Development and Education, University of Amsterdam, Amsterdam, The Netherlands

**Keywords:** Mindful parenting, Adolescents, Type 1 diabetes mellitus, Glycemic control, Quality of life

## Abstract

The objective of this study was to examine associations between the mindful parenting style of parents of adolescents (aged 12–18) with type 1 diabetes mellitus (T1DM), and the glycaemic control and quality of life (QoL) of the adolescents. Chronic health conditions, such as T1DM, that require demanding treatment regimens, can negatively impact adolescents’ quality of life. Therefore, it is important to determine whether mindful parenting may have a positive impact in these adolescents. Age, sex and duration of T1DM were examined as potential moderators. Parents (*N* = 215) reported on their own mindful parenting style (IM-P-NL) and the adolescents’ glycaemic control. Parents and the adolescents with T1DM (*N* = 129) both reported on adolescents’ generic and diabetes-specific QoL (PedsQL™). The results showed that a more mindful parenting style was associated with more optimal hemoglobin A_1c_ (HbA_1c_) values for boys. For girls, a more mindful parenting style was associated with not having been hospitalized for ketoacidosis. For both boys and girls, a more mindful parenting style was associated with better generic and diabetes-specific proxy-reported QoL. In conclusion, mindful parenting style may be a factor in helping adolescents manage their T1DM. Mindful parenting intervention studies for parents of adolescents with T1DM are needed to examine the effects on adolescents’ glycaemic control and their quality of life.

## Introduction

The number of European children with type 1 diabetes mellitus (T1DM) is increasing with an overall annual increase of almost 4 % (Patterson et al. 2009). Two-thirds of the children and adolescents with T1DM have suboptimal levels of glycaemic control (hemoglobin A_1c_ (HbA_1c_) values above the target value of 7.5 % or 58 mmol/mol (American Diabetes Association [ADA] 2015), with adolescent girls who have T1DM for a longer time having the least optimal HbA_1c_ values (Rosenbauer et al. 2012). Suboptimal glycaemic control can coincide with diabetic ketoacidosis, which in turn increases the risk for long-term chronic complications, such as neuropathy, nephropathy and retinopathy (The Writing Team for the Diabetes Control and Complications Trial/Epidemiology of Diabetes Interventions and Complications Research Group 2002; Silverstein et al. 2005). Rosenbauer et al. (2012) reported a crude ketoacidosis event incidence rate (hyperglycaemia with pH value < 7.3 and/or hospital admission due to ketoacidosis) of 5.9 ± 1.5 per 100 youths with T1DM aged 14.6 ± 3.7 years. Hypoglycaemia can also occur regardless of average glycaemic control. In a large multi-centre trend analysis, Rosenbauer et al. (2012) found that severe hypoglycaemic events, requiring assistance and/or with loss of consciousness or seizures, had crude incidence rates of respectively 19.1 ± 0.29 and 4.0 ± 0.13 per 100 youths aged 14.6 ± 3.7 years.

Beside problems with glycaemic control, adolescents with T1DM, girls even more than boys, report problems concerning their diabetes-specific quality of life (QoL) (Nieuwesteeg et al. 2012). Adolescents report that their T1DM negatively impacts their day-to-day life, and they are, for example, less satisfied with important (social and family) relationships, they frequently worry about their condition and diabetes-related issues and they find that treatment regimen is demanding (Emmanouilidou et al. 2008; Faulkner 2003; Graue et al. 2003; Jafari et al. 2011; Kalyva et al. 2011; Namakura et al. 2010).

Although definitions of QoL vary widely, there is consensus about two central aspects. First, QoL should be regarded as a multi-dimensional construct incorporating at least three broad domains, including physical, mental and social functioning (Nieuwesteeg et al. 2012; van Steensel et al. 2012). Second, considering the subjective character, QoL should be assessed from the person’s perspective whenever possible (Upton et al. 2008). When assessing the QoL of children, studies seem to randomly utilize self-reports (e.g. Hartman et al. 2007; Petersen et al. 2009) or parent proxy-reports (e.g. Klassen et al. 2004; Tomlinson et al. 2011), whereas research consistently showed that self-reports and parent proxy-reports are not interchangeable (Theunissen et al. 1998; Upton et al. 2008). Studies show noticeable differences between self-reports and parent proxy-reports on child’s generic and health-related QoL, ranging from weak-to-moderate and high agreement (e.g. Cremeens et al. 2006; Theunissen et al. 1998; Upton et al. 2008). Parents of children with a chronic condition such as T1DM tend to report lower health-related QoL for their children than do the children themselves (e.g. Nieuwesteeg et al. 2012). This disagreement between children and parents indicates that parent proxy-reports cannot be substituted for child self-reports and vice versa. Because parent proxy-reports and self-reports on QoL are not interchangeable, and because it is the parent’s perception of the child’s health-related QoL that is important in utilization of healthcare services (Varni et al. 2001), it is most relevant to use both self- and proxy-reports.

Because adolescents with T1DM are at increased risk for suboptimal glycaemic control and impaired QoL, it is important to determine factors that can be influenced and can positively impact the glycaemic control and QoL of these adolescents. Mounting evidence suggests that the parenting style of parents of adolescents with T1DM is directly related to glycaemic control and QoL of these adolescents (e.g. Cameron et al. 2008; Jaser and Grey 2010; Shorer et al. 2011; Stoker Green et al. 2010; Wysocki et al. 2008). One aspect of parenting of which the associations with glycaemic control and QoL are unknown is mindful parenting. Parents who have a mindful parenting style listen to their children with full attention, accept themselves and their child without judgment, are aware of their own and the child’s emotions, regulate themselves in the parenting relationship and have compassion for themselves and their child (Duncan et al. 2009).

The parenting style of parents of adolescents with T1DM has been shown to influence glycaemic control and QoL of these adolescents in several ways and the aspect of mindful parenting might play an important role in these relationships. One-way parenting style can influence the glycaemic control, and QoL of these adolescents is through the way parents handle diabetes-related conflicts and negative communication about diabetes with their children, because this is associated with the glycaemic control and QoL of the adolescents (e.g. Anderson et al. 2002; de Wit et al. 2007; Weissberg-Benchell et al. 2009). Intervention studies showed an improvement in glycaemic control when conflict resolution skills of families improve (Neylon et al. 2013). Parents who have a mindful parenting style might be better able to handle the difficult situations of negative diabetes-related interactions because they show less automatic and over-reactive responses (responses that are colored by, for example, experiences from the past and cognitive biases) (Bögels et al. 2010; van der Oord et al. 2012) and better anger management (Coatsworth et al. 2010). To illustrate, when a child has a high blood glucose level, an automatic response could be to react angrily, thinking that the adolescent did not pay enough attention as he/she did before (experience from the past). A parent with a more mindful parenting style will take time to consider if another explanation for the high blood glucose level is possible e.g. a stressful event for the adolescent, thus avoiding automatic negative interactions about the diabetes. In a recent study on parenting of young adolescents (12–14 years old), Turpyn and Chaplin (2015) found a positive association between parents reporting a more mindful parenting style, and less parental negative emotion (e.g. furrowed brows, crying) and greater shared positive emotion (e.g. looking at each other and laughing at the same time) during an observed parent–adolescent conflict interaction.

Another example of how parenting style can influence the glycaemic control and QoL of the adolescents is that parenting style is important when adolescents become more autonomous and the responsibilities for the demanding diabetes management tasks have to be renegotiated. Age-appropriate shared responsibility between parents and adolescents is related to more optimal glycaemic control and QoL (Helgeson et al. 2008; Silverstein et al. 2005; Weissberg-Benchell et al. 2009; Wiebe et al. 2005; Wysocki et al. 2009), and intervention studies enhancing parental involvement in the diabetes management showed improved glycaemic control (Neylon et al. 2013). In the challenge of renegotiating responsibility for the diabetes management, parents with a more mindful parenting style might be better able to come to shared responsibilities. Parents that report more mindful parenting have better parent–child communication (Duncan et al. 2015). Also, because they are more aware of their own cognitive biases around perceived and actual vulnerability of their child, unnecessary worry decreases (Duncan et al. 2009; Minor et al. 2006), likely reducing the risk for overcontrolling by the parent. In line with this idea, Bögels et al. (2014) found that a mindful parenting program, focusing on parenting in general, decreased parental overcontrol and had positive effects on autonomy encouraging parenting.

So far, regarding mindful parenting, no studies focused specifically on the mindful parenting of children or adolescents with (chronic) health conditions. On the effects of mindful parenting in other conditions, only preliminary experimental studies and studies with small sample sizes (*n* < 25) (e.g. Bögels et al. 2010; Sawyer Cohen and Semple 2010) have been published, except for one recent quasi-experimental study of Bögels et al. (2014) (*n* = 86). Yet, the first studies report that mindful parenting interventions improve parent and child interactions in conditions that have an impact on family functioning such as developmental disorders (e.g. attention-deficit/hyperactivity disorder (ADHD), autism) (e.g. Bögels et al. 2010; Sawyer Cohen and Semple 2010). Studies showed that mindful parenting can be improved by an intervention (Bögels et al. 2014; Coatsworth et al. 2010), and Coatsworth et al. (2010) found that when mindful parenting principles were added to a family intervention, there was a stronger improvement of the quality of the parent–adolescent relationship than without these principles (small to medium effects). Thus, mindful parenting could have a (additional) positive influence supplementing the current, mostly behaviour focused, interventions.

Mindful parenting thus seems to be a factor that could be associated with the glycaemic control and QoL of adolescents with T1DM, and a factor that can be influenced with interventions. The aim of the present study was to examine the associations between the self-reported mindful parenting style of parents and (1) glycaemic control, and (2) QoL of adolescents with T1DM, as reported by the adolescents themselves and their parents. We also examined how age and sex of the adolescents and duration of T1DM were associated with these relationships.

## Method

### Participants

Adolescents (aged 12–18 years) with T1DM (self-reported) and one parent (parents themselves chose which one), with sufficient mastery of the Dutch language, were included. There were no exclusion criteria. Because participants were (partially) recruited via a magazine, there is no information about the characteristics of the non-respondents. Of the large data bank of the Diabetes MILES study, we included the 215 parents who completed at least the Interpersonal Mindfulness in Parenting scale (IM-P-NL). We also included 129 (60 %) of their adolescent children who completed the QoL questionnaires (Pediatric Quality of life Inventory (PedsQL) 3.2™ Generic Core Scales and Diabetes Module).

Table 1 shows the socio-demographic and diabetes-related characteristics of the participants. The mean HbA_1c_ value of the adolescents (*M* 8.0 %, *SD* 1; *M* 63 mmol/mol, *SD* 12) was significantly higher than the norm of 7.5 % or 58 mmol/mol (ADA 2015) (*t*(186) = 5.17, *p* < .001) and only 32 % of the adolescents had HbA_1c_ values lower than 7.5 % or 58 mmol/mol. Overall, parents reported a similar mindful parenting style compared to the mothers of adolescents from the general community and compared to mothers of another sample of adolescents with T1DM (de Bruin et al. 2014). The generic and diabetes-specific self- and parent proxy-reported QoL were also comparable to those found in other samples of adolescents with T1DM and their parents (Engelen et al. 2009; Varni et al. 2003).

**Table 1:** Socio-demographic and diabetes-related characteristics of the participants

	*N*	%
Information provided by parent	215	
Parent is the mother	182/215	85 %
Parent is married/cohabiting	188/215	87 %
Parent has intermediate or higher vocational education	182/215	85 %
Adolescent is male	121/215	56 %
Treatment of adolescent T1DM with insulin pump	152/215	71 %
>0 Severe hypoglycaemic event in past 12 months for adolescent	26/213	12 %
>0 Hospitalization for ketoacidosis in last 12 months for adolescent	31/213	14 %
>0 Hospitalization related to T1DM in last 12 months for adolescent	32/215	15 %
Information provided by adolescents	129	
Male	67/129	52 %
Lower general secondary education	26/94	28 %
Higher general secondary education	26/94	28 %
Pre-university education	13/94	14 %
The Netherlands as country of birth mother	120/126	95 %
The Netherlands as country of birth father	121/125	97 %
The Netherlands as country of birth adolescent	126/126	100 %
	Mean (SD)	Min–max
Adolescents age (years)	14 (2)	12–18
Duration of T1DM (years)	6 (4)	0–18
Number of daily insulin injections	2 (2)	0–9
Number of daily glucose monitoring	5 (2)	1–10
HbA_1c_ (%)	8 (1)	5–11
HbA_1c_ (mmol/l)	63 (12)	33–97
One sample *t* test comparing mean HbA_1c_ with norm score of 7.5 % or 58 mmol/mol	t (186) = 5.17 p < .001	
	Mean (SD)	Min–max
IM-P-NL total score	106.25 (10.21)	136.00–73.00
Generic QoL total score parent proxy-report	76.28 (13.84)	100.00–36.41
Diabetes-specific QoL total score parent proxy-report	68.14 (13.90)	99.33–35.50
Generic QoL total score adolescent self-report	80.72 (12.23)	100.00–44.69
Diabetes-specific QoL total score adolescent self-report	73.43 (14.13)	100.00–31.00

*T1DM* Type 1 diabetes mellitus, *HbA*_*1c*_ hemoglobin A_1c_ value, *IM-P-NL* Interpersonal Mindfulness in Parenting scale, Dutch version, *QoL* quality of life

### Procedure

This cross-sectional study is part of Diabetes Management and Impact for Long-term Empowerment and Success (MILES)—The Netherlands, a national, online survey of people with diabetes. The rationale and methods of this large-scale study have been published elsewhere (Nefs et al. 2012). The study was approved by the Ethics Review Board of Tilburg School of Social and Behavioral Sciences (TSB) and was performed according to the Helsinki Declaration on human research.

Adolescents (aged 12–18 years) with T1DM and parents of adolescents with T1DM were invited to complete an online questionnaire by means of an announcement in the monthly magazine of the Dutch Diabetes Association (DDA). Parents of adolescents under 16 years of age could leave the e-mail address of their child on the registration page, and the child then received a link to the online assessment tool. Thus, the child could only participate after the parent had given his or her consent. The medical ethical rules state that adolescents of ≥16 years do not require parental consent to participate and could leave their e-mail address on the registration page themselves. In addition, adolescents and parents who are members of the DDA, and adolescents and parents from Diabeter (a national centre for paediatric and adolescent diabetes care and research) were approached by e-mail. In this e-mail invitation, adolescents and parents were asked to complete the online survey by visiting the study-specific website and register themselves by giving their e-mail address.

### Measures

#### Mindful Parenting

The parent reported on his/her mindful parenting style with the Interpersonal Mindfulness in Parenting scale, Dutch version (IM-P-NL) (de Bruin et al. 2014; Duncan 2007). This self-report questionnaire measures affective, cognitive and attitudinal aspects of parent–child relationships. Each of the 29 items is scored on a 5-point Likert scale, ranging from 1 = ‘never true’ to 5 = ‘always true’. Higher subscale scores reflect a higher degree of mindfulness in parenting. The questionnaire has six subscales: listening with full attention, compassion for the child, non-judgmental acceptance of parental functioning, emotional non-reactivity in parenting, emotional awareness of the child, emotional awareness of self, and a total score. We used the total score (internal consistency for the current sample was *α* = .85). The IM-P-NL has good psychometric properties in a Dutch sample of mothers of adolescents and is also valid in a Dutch sample of mothers of adolescents with T1DM (de Bruin et al. 2014).

#### Glycemic Control

The value and date of the latest HbA_1c_ measure in percentage and mmol/mol were reported by the parents. HbA_1c_ values indicate the average blood glucose level over the past 2–3 months and are routinely measured quarterly. The goal for adolescents is an HbA_1c_ value below 7.5 % or 58 mmol/mol (ADA 2015). Higher values indicate a less optimal glycaemic control. In addition, we asked parents to provide the number of severe hypoglycaemic events, hospitalizations for ketoacidosis and hospitalizations related to T1DM in general, all in the last 12 months. These scores were dichotomized with a score of 0 meaning that the event did not occur and a score of 1 meaning that the event occurred at least once in the last 12 months.

#### Quality of Life

Generic QoL was assessed with the adolescent Dutch version of the Pediatric Quality of life Inventory (PedsQL) 3.2™ Generic Core Scales (Varni et al. 2003) and by proxy using the parent version. The 23 items are scored on a 5-point Likert scale ranging from 0 = ‘never’ to 4 = ‘almost always’, higher scores indicating a better QoL. We used the total scores (internal consistency for the current sample was *α* = .87 for adolescents and *α* = .90 for parents). This questionnaire proved to have adequate psychometric properties in a sample of children and adolescents with T1DM (Varni et al. 2003) and in Dutch children and adolescents, including those with a chronic condition (Engelen et al. 2009).

Diabetes-specific QoL was assessed with the adolescent version of the Pediatric Quality of Life Inventory (PedsQL) 3.2™ Diabetes Module (Varni et al. 2003) and by proxy using the parent version. Each was translated specifically for this study via backward and forward translation with permission from the author. The 32 items were scored on the same Likert scale as the PedsQL 3.2™ Generic Core Scales. We used the total scores (internal consistency for the current sample was *α* = .91 for adolescents and *α* = .92 for parents). The original version has adequate reliability in a sample of adolescents with T1DM (Varni et al. 2003).

#### Demographic and Diabetes-Related Characteristics

The parent was asked to report his/her relation to the adolescent, marital status and educational level. He/she indicated whether the T1DM was treated with insulin injections or an insulin pump and reported the number of daily glucose monitoring and insulin injections (when relevant). The adolescent provided his/her sex, educational level, country of birth and country of birth of both parents.

### Data Analyses

Frequencies and descriptive statistics were used to present the diabetes-related and socio-demographic characteristics of the participants. A one-sample *t* test was used to compare the mean HbA_1c_ value to the norm of 7.5 % or 58 mmol/mol (ADA 2015).

Missing values for the QoL and mindful parenting measures were imputed with the mean of the scale score if 50 % or more of the items were completed (Varni et al. 2001). Pearson correlation coefficients were calculated to examine the associations between the continuous variables: mindful parenting, and adolescents’ glycaemic control (HbA_1c_ values), adolescent-rated generic and diabetes-specific QoL, and parent proxy-reported generic and diabetes-specific QoL. For the dichotomous variables (number of severe hypoglycaemic events, hospitalizations for ketoacidosis and hospitalizations related to T1DM in general), Spearman’s rho was used.

Subsequently, regression analyses were used to assess whether mindful parenting was associated with adolescents’ glycaemic control and QoL variables, with sex and age of the adolescents, and the duration of their T1DM examined as interaction variables (according to the method described in Twisk 2010). For the continuous variables (HbA_1c_ values, adolescent-rated generic and diabetes-specific QoL and proxy-reported generic and diabetes-specific QoL), linear regression analyses were used (method: enter). For the dichotomous variables (number of severe hypoglycaemic events, hospitalizations for ketoacidosis and hospitalizations related to T1DM in general), we used logistic regression analyses (method: enter).

## Results

Table 2 illustrates that adolescents with better proxy-reported generic and diabetes-specific QoL tended to have a parent with a more mindful parenting style (respectively *r* = .29, *p* < .01; *r* = .34, *p* < .001). No other significant correlations with mindful parenting were found.

**Table 2:** Correlations (Pearson *r* and Spearman’s *rho*) examining associations between mindful parenting, glycaemic control, and adolescent- and proxy-reported QoL

	Parent self-report	Parent proxy-report				Parent proxy-report		Adolescent self-report	
	Mindful parenting	Glycemic control				QoL		QoL	
	IM-P-NL total score	HbA1c	Severe hypoglycaemic events	Hospitalization for ketoacidosis	Hospitalization related to T1DM in general	Generic QoL total score	Diabetes-specific QoL total score	Generic QoL total score	Diabetes-specific QoL total score
Reported by parent									
Mindful parenting IM-P-NL total score	–	−.11^a^	−.05^b^	−.12^b^	−.05^b^	.29**^a^	.34***^a^	.15^a^	.17^a^
HbA1c		–	−.04^b^	.19*^b^	.01^b^	−.21*^a^	−.38***^a^	−.17^a^	−.22*^a^
Severe hypoglycaemic events			–	.10^b^	.13^b^	−.11^b^	−.16*^b^	−.13^b^	−.19*^b^
Hospitalization for ketoacidosis				–	.27**^b^	−.16^b^	−.27**^b^	−.31**^b^	−.19*^b^
Hospitalization related to T1DM in general					–	−.14^b^	−.15*^b^	−.26*^b^	−.18^b^
Generic QoL total score						–	.72***^a^	.64***^a^	.51***^a^
Diabetes-specific QoL total score							–	.48***^a^	.61***^a^
Reported by adolescent									
Generic QoL total score								–	.71***^a^
Diabetes-specific QoL total score									–

*IM-P-NL* Interpersonal Mindfulness in Parenting scale, Dutch version, *HbA*_*1c*_ hemoglobin A_1c_ value, *T1DM* type 1 diabetes mellitus, *QoL* quality of life

*p < .05; **p < .01; ***p < .001

^a^Pearson *r*

^b^Spearman’s rho

Sex had a moderation effect on the association between mindful parenting and the HbA_1c_ values of the adolescents: Among boys, but not girls, higher mindful parenting style scores were associated with lower HbA_1c_ values (*β* = −0.22, *p* < .05, 95 % CI [−0.04, −0.00]; *F*(101) = 5.23, *p* = .02) (Table 3). Sex also moderated the association between the mindful parenting style of the parent and the adolescent hospitalization for ketoacidosis in the last 12 months: Girls, but not boys, who had not been hospitalized for ketoacidosis, had parents who reported a more mindful parenting style (OR = 0.92, *p* < .05, 95 % CI [0.86, 0.99]; *χ*^2^ (1, *N* = 94) = 6.03, *p* = .01).

**Table 3:** Regression analyses predicting glycemic control from mindful parenting, with age, sex and duration of T1DM as interaction variables, all reported by parent

Predictor variables Reported by parent	HbA1c^†^ *β [95% CI]*		Severe hypoglycemic events^††^ *OR [95% CI]*	Hospitalization for ketoacidosis^††^ *OR [95% CI]*		Hospitalization related to T1DM in general^††^ *OR [95% CI]*
Mindful parenting IM-P-NL Total score	−0.11 [−0.03,0.00]		0.98 [0.95,1.03]	0.98 [0.94,1.01]		0.99 [0.95,1.03]
Mindful parenting IM-P-NL Total score	−0.11 [−0.04,0.01]		1.00 [0.93,1.08]	1.01 [0.93,1.11]		1.00 [0.95,1.07]
Duration of T1DM	0.01 [−0.41,0.41]		1.44 [0.46,4.52]	2.04 [0.63,6.60]		1.40 [0.46,4.23]
Mindful parenting x Duration of T1DM	0.32 [−0.00,0.00]		1.00 [0.99,1.01]	1.00 [0.98,1.01]		1.00 [0.99,1.01]
Mindful parenting IM-P-NL Total score	0.57 [−0.07,0.18]		0.69 [0.47,1.01]	0.78 [0.56,1.09]		1.10 [0.80,1.54]
Age of adolescent	0.92 [−0.35,1.47]		0.11 [0.01,1.52]	0.22 [0.02,2.40]		2.73 [0.26,28.94]
Mindful parenting x Age of adolescent	−0.08 [−0.01,0.00]		1.02 [1.00,1.05]	0.19 [0.99,1.04]		0.99 [0.97,1.02]
Mindful parenting IM-P-NL Total score	−0.21 * [−0.04,-0.00]		1.01 [0.95,1.07]	0.92 * [0.86,0.99]		0.99 [0.92,1.05]
Sex of adolescent	−1.52 [−6.42,0.06]		36.18[0.01,241496.28]	0.00 * [0.00,0.26]		0.44 [0.00,1777.91]
Mindful parenting x Sex of adolescent	1.61 * [0.00,0.06]		0.96 [0.88,1.04]	1.10 * [1.01,1.20]		1.01 [0.93,1.09]
	Male adolescents	Female adolescents		Male adolescents	Female adolescents	
Mindful parenting IM-P-NL Total score	−0.22 * [−0.04,-0.00]	0.10 [−0.01,0.03]		1.01 [0.96,1.01]	0.92 * [0.86,0.99]	
Mindful parenting IM-P-NL Total score	−0.16 [−0.04,0.01]	0.19 [−0.03,0.07]		1.07 [0.95,1.19]	0.88 [0.75,1.04]	
Duration of T1DM	0.50 [−0.45,0.71]	0.78 [−0.44,0.84]		2.21 [0.43,11.31]	0.84 [0.13,5.28]	
Mindful parenting x Duration of T1DM	−0.06 [−0.01,0.00]	−0.64 [−0.01,0.00]		1.00 [0.98,1.01]	1.00 [0.99,1.02]	
Mindful parenting IM-P-NL Total score	0.48 [−0.11,0.20]	1.02 [−0.09,0.32]		0.89 [0.56,1.42]	0.48 * [0.25,0.94]	
Age of adolescent	0.73 [−0.70,1.67]	1.54 [−0.63,2.31]		0.37 [0.01,12.90]	0.02 [0.00,1.26]	
Mindful parenting x Age of adolescent	−1.01 [−0.02,0.01]	−1.72 [−0.02,0.01]		1.01 [0.98,1.04]	1.04 [1.00,1.09]	

† Linear regression analyses †† Logistic regression analyses

*p < .05; ** *p* < .01; ***p < .001

*Note*. T1DM = Type 1 Diabetes Mellitus; HbA1c = Hemoglobin A1c value; IM-P-NL = Interpersonal Mindfulness in Parenting scale, Dutch version

With regard to the associations between the mindful parenting style of the parent and QoL, Table 4 shows that more mindful parenting was associated with parental reports of better generic QoL for the adolescents (*β* = 0.29, *p* < .01, 95 % CI [0.15, 0.66]; *F*(112) = 10.17, *p* = .002), but this association was not found with adolescent-rated generic QoL. More mindful parenting was also associated with parental reports of better diabetes-specific QoL for the adolescents (*β* = 0.34, *p* < .001, 95 % CI [0.28, 0.63]; *F*(213) = 27.03, *p* < .001), but not with adolescent-rated diabetes-specific QoL. There were no moderation effects found for sex and age of the adolescents, or for the duration of their T1DM on the association between the mindful parenting style of the parent and QoL (adolescent- or proxy-reported).

**Table 4:** Regression analyses predicting self- and proxy-reported QoL from mindful parenting with age, sex and duration of T1DM as interaction variables

	Generic QoL total score	Diabetes-specific QoL total score	Diabetes-specific QoL total score	Generic QoL total score
Predictor variables	Parent proxy-report	Parent proxy-report	Adolescent self-report	Adolescent self-report
Reported by parent	*β* [95 % CI]	*β* [95 % CI]	*β* [95 % CI]	*β* [95 % CI]
Mindful parenting IM-P-NL total score	0.29** [0.15, 0.66]	0.34*** [0.28, 0.63]	0.17 [−0.02, 0.51]	0.15 [−0.07, 0.44]
Mindful parenting IM-P-NL total score	0.22 [−0.15, 0.77]	0.38** [0.22, 0.80]	0.21 [−0.16, 0.78]	0.08 [−0.36, 0.55]
Duration of T1DM	−0.55 [−8.69, 5.05]	0.29 [−3.93, 5.84]	0.27 [−5.92, 7.69]	−0.60 [−8.19, 4.83]
Mindful parenting × duration of T1DM	0.51 [−0.05, 0.08]	−0.42 [−0.06, 0.03]	−0.45 [−0.08, 0.05]	0.53 [−0.05, 0.08]
Mindful parenting IM-P-NL total score	0.27 [−2.05, 2.81]	−0.03 [−1.51, 1.43]	0.17 [−2.22, 2.71]	0.12 [−2.63, 2.91]
Age of adolescent	0.16 [−16.07, 19.00]	−0.61 [−15.80, 5.83]	−0.20 [−19.76, 16.21]	0.07 [−19.33, 20.53]
Mindful parenting × age of adolescent	0.02 [−0.16, 0.17]	0.58 [−0.07, 0.14]	0.02 [−0.17, 0.17]	0.05 [−0.18, 0.19]
Mindful parenting IM-P-NL total score	0.34** [0.15, 0.80]	0.39*** [0.33, 0.74]	0.26* [0.08, 0.70]	0.24 [−0.02, 0.61]
Sex of adolescent	0.78 [−32.33, 75.23]	0.85 [−14.87, 62.24]	1.47 [−14.18, 97.18]	1.26 [−24.81, 85.99]
Mindful parenting × sex of adolescent	−0.98 [−0.76, 0.25]	−1.07 [−0.64, 0.08]	−1.79 [−0.99, 0.05]	−1.41 [−0.84, 0.20]

*QoL* quality of life, *T1DM* type 1 diabetes mellitus, *IM-P-NL* Interpersonal Mindfulness in Parenting scale, Dutch version

*p < .05; **p < .01; ***p < .001

## Discussion

The aim of the present study was to examine the associations between the self-reported mindful parenting style of the parents and glycaemic control and self- and proxy-reported QoL of adolescents with T1DM. We also examined whether age and sex of the adolescents and duration of T1DM moderated these associations. Because adolescents with T1DM are at increased risk for suboptimal glycaemic control and impaired QoL, it is important to study and determine factors that can impact positively on the glycaemic control and QoL of these adolescents and that can be influenced with interventions. In the present study, we explored whether mindful parenting could be such a factor. To our knowledge, these associations have not been examined before. The results of the present study suggest that the mindful parenting style of the parents is related to more optimal glycaemic control in adolescent boys (i.e. HbA_1c_), to adolescent girls not having been hospitalized for ketoacidosis in the last 12 months and to proxy-reported generic and diabetes-specific QoL of both male and female adolescents with T1DM (though not to generic or diabetes-specific QoL as rated by the adolescents themselves).

In the present study, more than two-thirds of the adolescents had suboptimal glycaemic control, which is in line with a recent large-scale European study (Rosenbauer et al. 2012). Boys of parents with a more mindful parenting style had more optimal (lower) HbA_1c_ values. These results are striking because, specifically, adolescent boys with T1DM report worse family relations (Leonard et al. 2002), while our results seem to suggest the potential importance of a good parent–adolescent relationship for boys with T1DM. For girls, a more mindful parenting style of the parent was related to less likelihood of having been hospitalized for ketoacidosis in the last 12 months. Adolescent girls who have had T1DM for a longer period have more episodes of ketoacidosis in a year than boys (7.3 ± 0.5 vs. 5.8 ± 0.2; *p* = .03; Fritsch et al. 2011), and Rosenbauer et al. (2012) found that adolescent girls who have T1DM for a longer period are more at risk for suboptimal glycaemic control. Our results, mindful parenting being associated with more optimal glycaemic control in adolescent boys and with adolescent girls not having been hospitalized for ketoacidosis in the last 12 months, suggest that a more mindful parenting style could help adolescent boys and girls to manage their T1DM. That the relationship between mindful parenting and glycaemic control was different for boys and girls might be a result of girls reaching adolescent developmental tasks of separation and individuation earlier than boys (Dashiff 2001; Petersen and Leffert 1995) and of the expectancy that adolescent girls manage their T1DM more independently, while this is less expected of boys (Schmidt 2007; Williams 1999), potentially leading to parenting style having a more direct influence on boys’ control over their glycaemic control and influencing girls only in the case of more overt deregulation of the T1DM.

In the present study, we found no significant associations between mindful parenting and generic or diabetes-specific QoL as rated by the adolescents themselves. Because of the subjective character of QoL, it is often recommended to measure this construct by means of a self-report measure, but assessment of the parent’s perceptions of their child’s QoL could contribute to a more complete understanding of the childs’ QoL (Upton et al. 2008). In the present study, parents that reported having a more mindful parenting style also reported their adolescents having a better generic and diabetes-specific QoL. It is possible that other factors (such as social desirability or how parents themselves are affected by the T1DM) explain parents scoring higher for both mindful parenting and their perception of their child’s QoL. The study by Cremeens et al. (2006) showed that parents’ own QoL is significantly correlated to their proxy-report of their child’s QoL. It may be the case that parents who have a more mindful parenting style also have a better QoL themselves and therefore report more positively about the QoL of their children.

The significant relationships between mindful parenting, glycaemic control and hospitalization due to ketoacidosis found in the present study, suggest that improving mindful parenting in the parents potentially may benefit adolescents with T1DM. It may be families of children with other chronic conditions (like e.g. asthma, cancer) that could also benefit from a mindful parenting style, because though they vary from T1DM from a clinical/medical point of view, these conditions also have considerable impact on the family unit, in causing stress and concern and the need for intensive treatment regimens (e.g. Cousino and Hazen 2013). In a systematic review, Salema et al. (2011) found that involving the family in chronic care for the adolescents is one of the features that make interventions effective. Mindful parenting could have a (additional) positive influence supplementing the current interventions (Coatsworth et al. 2010), or as a stand-alone mindful parenting course (Bögels and Restifo 2013). Because, in adolescents with T1DM, diabetes-specific parent–child variables have a stronger relation with QoL and treatment adherence than generic parent–child variables (Ellis et al. 2007; Weissberg-Benchell et al. 2009), an intervention that also directly targets diabetes-specific parent–child interactions might have the most positive result. In such an intervention, parents for example would learn to think in terms of ‘high values’ instead of the automatic thinking of ‘bad values’ when they see an above target blood glucose reading from their child.

This study has several limitations, many of which have been reported elsewhere (Nefs et al. 2012). Because of internet-based data collection, metabolic control was assessed by a self-report measure. An explanation for not detecting relations between mindful parenting and some domains of metabolic control could be that parents did not reliably report their child’s metabolic control. Consequently, associations between mindful parenting and some domains of metabolic control could have remained undetected. More objective measures, such as consulting hospital records for clinical data, may give more accurate results. Also, mindful parenting was reported by parents themselves, whereas parenting may have been more reliably assessed by observations. However, Duncan et al. (2015) found preliminary evidence that there is an association between self-reported mindful parenting and observed parent–youth interaction, making the use of self-report on mindful parenting defendable. Also, parent respondents in this study were most likely to be highly educated mothers (Table 1), which is a clear limitation. Given the under-representation of fathers and respondents with lower education levels, results cannot be generalized to these groups without caution.

Further, because associations found in this study were small to medium, and some non-significant results had large confidence intervals, the clinical significance needs to be examined critically. It could be argued that even though the relationships found in this study were small to medium, a factor such as mindful parenting, which can be influenced by an intervention, should be thoroughly studied if it could have a positive influence on the outcome of the chronic disease T1DM. Glycaemic control, after all, is one of the predictors of the development of complications in T1DM (The Writing Team for the Diabetes Control and Complications Trial/Epidemiology of Diabetes Interventions and Complications Research Group 2002), and QoL is also an important outcome variable (Matza et al. 2004).

This study was not designed to test the existence of a causal relationship between the mindful parenting of parents and the glycaemic control and QoL of adolescents with T1DM. The next steps could be a longitudinal follow-up study to establish temporal order of the variables, as well as a controlled intervention study in which the mindful parenting style of parents of adolescents with T1DM is increased through a mindful parenting course. It is possible that the positive relationships between mindful parenting and glycaemic control (in boys), hospitalization due to ketoacidosis (in girls), and proxy-reported generic and diabetes-specific QoL of the adolescents found in this study are not caused by the mindful parenting style positively influencing the outcome variables but rather the other way around. It is possible that parents may experience less stress when their children experience more optimal glycaemic control and when they perceive their children to have better QoL, and therefore, these parents might be better able to use a mindful parenting style. However, longitudinal and intervention studies thus far support the idea that parent support and parental involvement in the diabetes care of their children are important for adolescents in managing their T1DM (Salema et al. 2011) and have a positive relation with adolescent-reported QoL (Skinner et al. 2000), suggesting that parenting style might positively influence glycaemic control and QoL of the adolescents.

As a last limitation, we want to report that this cross-sectional study was not designed to identify the mechanisms by which mindful parenting influences the outcome variables. We assert that particularly, the mechanism of lower automaticity (parents being able to not automatically react from, for example, experiences from the past and cognitive biases) may provide a starting point for exploring this issue.

In conclusion, parent-reported mindful parenting was directly related to more optimal glycaemic control in adolescent boys, not having been hospitalized for ketoacidosis in the last 12 months for adolescent girls, and with better perceived generic and diabetes-specific QoL of adolescents with T1DM (as rated by the parent but not by the adolescents themselves). A longitudinal follow-up study is needed to investigate the temporal order of the variables and to test the effectiveness of a mindful parenting training that includes interventions that directly target diabetes-specific parent–child interactions.
